# Altered white matter connectivity in children with congenital heart disease with single ventricle physiology

**DOI:** 10.1038/s41598-023-28634-9

**Published:** 2023-01-24

**Authors:** Brady J. Williamson, Maria E. Barnes-Davis, Jennifer Vannest, Julia S. Anixt, Haleh C. Heydarian, Lisa Kuan, Cameron S. Laue, Jayant Pratap, Mark Schapiro, Stephanie Y. Tseng, Darren S. Kadis

**Affiliations:** 1 Department of Radiology, University of Cincinnati, Cincinnati, OH USA; 2 Department of Neonatology, Cincinnati Children’s Hospital Medical Center, Cincinnati, OH USA; 3 Department of Pediatrics, University of Cincinnati, Cincinnati, OH USA; 4 Department of Communication Sciences and Disorders, University of Cincinnati, Cincinnati, OH USA; 5 Division of Developmental and Behavioral Pediatrics, Cincinnati Children’s Hospital Medical Center, Cincinnati, OH USA; 6 Cardiology, Cincinnati Children’s Hospital Medical Center, Cincinnati, OH USA; 7 Department Psychology, Pacific University, Forest Grove, OR USA; 8 Divisions of Cardiac Anesthesia and Cardiac Critical Care Medicine, Department of Anesthesia and Critical Care Medicine and Cardiac Center, Children’s Hospital of Philadelphia, Philadelphia, PA USA; 9 Neurology, Cincinnati Children’s Hospital Medical Center, Cincinnati, OH USA; 10 The Heart Center, Nationwide Children’s Hospital, Columbus, OH USA; 11 Neurosciences and Mental Health, Research Institute, Hospital for Sick Children, 686 Bay Street, Toronto, ON M5G 0A4 Canada; 12 Department of Physiology, University of Toronto, Toronto, ON Canada

**Keywords:** Cardiology, Neurology, Neurophysiology

## Abstract

Children born with congenital heart disease (CHD) have seen a dramatic decrease in mortality thanks to surgical innovations. However, there are numerous risk factors associated with CHD that can disrupt neurodevelopment. Recent studies have found that psychological deficits and structural brain abnormalities persist into adulthood. The goal of the current study was to investigate white matter connectivity in early school-age children (6–11 years), born with complex cyanotic CHD (single ventricle physiology), who have undergone Fontan palliation, compared to a group of heart-healthy, typically developing controls (TPC). Additionally, we investigated associations between white matter tract connectivity and measures on a comprehensive neuropsychological battery within each group. Our results suggest CHD patients exhibit widespread decreases in white matter connectivity, and the extent of these decreases is related to performance in several cognitive domains. Analysis of network topology showed that hub distribution was more extensive and bilateral in the TPC group. Our results are consistent with previous studies suggesting perinatal ischemia leads to white matter lesions and delayed maturation.

## Introduction

Individuals with single ventricle congenital heart disease (CHD) have seen a dramatic increase in survival thanks to novel diagnostics and surgical techniques, namely staged palliation with the Fontan circulation as an endpoint^1,2^. This staged cardiac reconstruction was introduced for children with single ventricle physiology and consists of three stages^3^: (1) an initial palliative procedure within the first few days of life that ensures that the single ventricle supplies both systemic and pulmonary circulations, (2) creation of a bi-directional Glenn anastomosis between 2 and 6 months, and (3) completion of the Fontan circuit by detaching the inferior vena cava from the heart and anastomosing it directly to the pulmonary artery between 1.5 and 3 years^4^.

Until this final stage is completed, systemic arterial oxygen saturation is considerably reduced compared to fully saturated healthy individuals (typically 75% after the first stage and 85% after the second). Improved survival of patients with CHD has led to awareness of long-term neurodevelopmental deficits, which are among the most common and potentially debilitating outcomes for patients with CHD^5^. Individuals with single ventricle conditions who have undergone neonatal palliation are among those with CHD at the highest risk for neurodevelopmental disabilities^6^.

There are several risk factors for neurodevelopmental delays and injury in this population, including alterations in blood flow and oxygen delivery, exposure to anesthetic drugs, cardiopulmonary bypass, extracorporeal membrane oxygenation (ECMO), possible cardiac arrest, prolonged mechanical ventilation and hospitalization, congenital central nervous system abnormalities, and chromosomal or genetic abnormalities^7–16^. Prolonged perioperative hypoxemia is specifically associated with attentional and executive deficits and may be responsible for damage in the oxygen sensitive regions of the prefrontal cortex^8,17,18^.

Brain abnormalities associated with neurodevelopmental deficits have been noted across the lifespan for individuals with CHD^7,19–23^. White matter injury (WMI) is the most common, affecting infants with CHD three times as often as controls^24^. Children with CHD requiring open heart surgery are 15 times more likely to have a brain abnormality than term comparisons^25^. These abnormalities are associated with impairments in IQ, attention, and motor skills^8,25–27^.

A study investigating cognitive functioning in preschool children following the Fontan procedure found that survivors have low-average performance but, in most cases without comorbid genetic disorders, are not severely impaired^28^. Studies of children who have undergone surgery requiring cardiopulmonary bypass in the neonatal period have found increased risk for motor and behavioral problems versus term children or very preterm children without CHD^29,30^. Domains that have been deemed at higher risk are visuomotor integration, processing speed, and executive functioning, including inattention, hyperactivity, and behavioral problems^15,18,26,28,31–34^. Increased risk of deficits in these domains persists throughout childhood and into adolescence. Despite the high prevalence of WMI in CHD, few studies have looked at long-term sequelae of these injuries using modern diffusion imaging techniques beyond infancy.

Diffusion weighted magnetic resonance imaging (dMRI) is commonly used to investigate white matter integrity and connectivity by measuring the flow of water. Conventionally, water flow within the voxel has been modeled as a 3D ellipsoid (tensor), the shape of which represents the diffusion distance in each direction^35,36^. Fractional anisotropy (FA) characterizes the strength of diffusion in the long axis of the ellipsoid (primary direction) relative to the secondary and tertiary axes^36^. This approach combines the intracellular and extracellular compartments in the voxel and is based on the perceived shape of myelinated axons. DTI studies in school-age children and adolescents who have undergone cardiac surgery for CHD have shown widespread reduction of FA compared to healthy controls and have related these changes to decreased performance in a variety of cognitive domains, including verbal memory, auditory attention, mathematical reasoning, processing speed, full-scale IQ, and visuomotor ability^32,34,37,38^.

Alternately, flow within a voxel can be modeled as (at least) two compartments: 1) the intracellular compartment, which is thought to be anisotropic as the diffusion is restricted by myelin, cell membranes, and other intracellular components, and 2) the extracellular compartment, which is thought to be isotropic and represents the extracellular environment. Using these higher-order models, such as generalized q-sampling imaging (GQI^39^), it is possible to derive more than one fiber direction within each voxel (i.e., incoherent fibers) that are thought to occur in up to 90% of white matter^40^. Additionally, these previous studies use a region-of-interest (ROI) based approach or an approach in which white matter is “skeletonized” to only include the central most part of white matter tracts with the highest FA. Novel dMRI acquisitions and analyses have recently been developed to overcome these limitations^39,41–44^.

There is a relative paucity of studies using higher-order diffusion models to characterize white matter development in infants and children with CHD^24,45^. To our knowledge, only two such studies, have been published. Using DTI, neurite orientation and distribution diffusion imaging (NODDI), and Gaussian Mixture Modeling (GMM), Karmacharya and colleagues showed that all three methods revealed consistent and expected changes in white matter in neonates born with CHD, but GMM exhibited the largest effect sizes^46^. Another group, using similar methods, found lower neurite density in association tracts and in the corpus callosum^47^. However, these studies used ROI-based approaches, averaging over regionally variant effects along the tracts examined.

Tractography derived from dMRI can also be used to investigate structural connectivity in the brain. Structural connections between regions can be represented by a matrix where each entry represents a connection between two regions. These connectivity matrices can then be passed on to graph theoretical analyses to assess the global and local topography of the network^48^. Nearly all studies to this point that have utilized this technique to investigate either structural or functional connectivity in children with CHD have been focused on neonates. These studies have found delayed network maturation, reduced regional connectivity, increased small worldness (a measure of the balance between network segregation and integration), decreased global efficiency (a measure of network integration), and higher network transitivity (a measure of network segregation)^49–54^.

To date, there have only been two studies that have considered brain structural connectivity aberrations in children born with CHD that persist into later childhood. These studies found differences global efficiency and small-worldness between CHD patients and healthy controls that were correlated with worse cognitive performance^55^. However, it is important to note that these were not the same CHD pathology that is being investigated here. A natural extension to these previous studies on global topology is to look at the distribution of the most important regions, called “hubs”, in children born with CHD versus healthy controls.

In the current study, we employ GQI to reconstruct the diffusion signal in each voxel and calculate quantitative anisotropy (QA^56^) based on this reconstruction. QA is a measure of diffusivity that is also weighted by the density of the diffusing molecules in a certain fiber direction. Previous studies have shown that QA is more accurate than FA and comparable to metrics derived from other higher-order models, but is much simpler and generalizable across diffusion imaging protocols^56^. QA values for each fiber direction within each voxel are then passed to a mass univariate, non-parametric regression framework, called group connectometry, to assess statistical differences in white matter within the cohort^57^.

The current study aims to characterize white matter differences between children born with CHD who have undergone Fontan palliation and heart-healthy, typically-developing controls (TPC), using a higher-order diffusion modeling approach that is sensitive to crossing fibers (GQI), and to implement voxel-wise statistical analyses sensitive to local effects along tracts (group connectometry). We will also assess whether performance on neuropsychological assessments is related to white matter connectivity within each group. Additionally, we will calculate graph theoretical measures for each group based on diffusion tractography to assess network topological differences between groups.

## Methods

### Participants

A total of 13 children with palliated single ventricle CHD, and 13 TPC, all between the ages of 6 and 11 years, were recruited for this study. Inclusion criteria for the CHD group were history of complex cyanotic heart disease, specifically single ventricle physiology, and completed Fontan circulation. Exclusion criteria for the CHD group were genetic syndrome associated with neurocognitive disability or delay, history of stroke, history of brain shunt, premature birth (< 36 weeks), cerebral palsy, epilepsy or other seizure disorder, significant visual or hearing impairment, movement disorder, and surgery within the last 6 months. Inclusion criteria for TPC were negative history for cardiovascular, neurologic, or psychiatric disorder and negative history for speech, language, and learning disability. Exclusion criteria were past, present, or anticipated special education placement, gestational age < 36 or > 42 weeks, and any chronic illness. This study was approved by the local Institutional Review Boards of the University of Cincinnati and Cincinnati Children’s Hospital Medical Center. Informed consent was obtained from the legal guardian of all participants. All study activities were carried out in accordance with the Declaration of Helsinki and all data analyzed was anonymized.

### Neuropsychological Evaluation and Demographics

A comprehensive neuropsychological battery was administered to participants including: all indices of the Wechsler Intelligence Scale for Children (WISC-V^58^), Trail Making, Verbal Fluency, and Color Word Interference from the Delis Kaplan Executive Function System (D-KEFS^59^), Peabody Picture Vocabulary Test (PPVT-4^60^), Expressive Vocabulary Test (EVT-2^61^), the Grooved Pegboard test^62^, and Behavior Rating Inventory of Executive Function (BRIEF^63^). Student’s *t*-tests were performed to test to test for differences between groups for all continuous variables and Fisher’s exact tests were performed for all categorical variables.

### MRI acquisition

All MRI data were collected in a single scanning session on a 3 T Philips Ingenia scanner. The MRI session consisted of a high-resolution T1-weighted sequence (TR/TE = 8.1/3.7 ms, flip angle = 8°, minimum inversion time = 940 ms, Matrix = 256 × 224, slices = 160, 1 × 1 × 1 mm resolution), a reverse phase encoded b0 scan to potentially use in preprocessing the dMRI data, a dMRI sequence consisting of 120 gradient directions at a b-value of 3000 and 5 b0 images (TR/TE = 3187/127 ms, Matrix = 112 × 112, slices = 72, 2 × 2 × 2 mm, SENSE = 1.2, MB = 4, Diffusion DELTA/delta = 64.3/40.2 ms), a high-resolution T2-weighted sequence (TR/TE = 2500/250 ms, Matrix = 256 × 224, slices = 200, 1 × 1 × 1 mm), and a second dMRI sequence with two b-values of 1000 and 2000 (30 and 60 gradient directions, respectively) and 5 b0s (TR/TE = 3187/127, Matrix = 112 × 112, slices = 72, 2 × 2 × 2 mm, SENSE = 1.2, MB = 4, Diffusion DELTA/delta = 64.3/40.2 ms). fMRI was also acquired for all participants but not used for the current analyses.

### Diffusion MRI preprocessing

Diffusion data were preprocessed using the TORTOISE toolbox^64^. First, T2-w images were skullstripped using HD-BET^65^. Then, the diffusion sequences were combined to obtain a final 3 shell (b = 1000, 2000, 3000) 210 direction (30, 60, 120) dataset that was used as input for the TORTOISE pipeline. Preprocessing included eddy current distortion correction, geometric distortion correction, motion correction, Gibbs ringing correction, and denoising. AFNI^66^ was then used to convert the TORTOISE b-matrix, properly adjusted/rotated for preprocessing steps, to a format compatible with DSI Studio (https://dsi-studio.labsolver.org/) for further analyses. Each output from TORTOISE was checked visually to ensure quality of preprocessing.

A study-specific template was constructed for group analyses. First, diffusion spin distribution functions (SDFs) were reconstructed using generalized q-sampling imaging^39^. Quantitative anisotropy (QA^56^) and isotropic diffusion (ISO) maps were exported from this reconstruction and used in template construction. Multivariate Template Construction from Advanced Normalization Tools (ANTs) was used to construct a group template for both QA and ISO^67^. These files were used as the template image for Q-Space Diffeomorphic Reconstruction (QSDR^68^). From these files, a connectometry database was constructed for statistical analyses. Quality was monitored throughout processing by (1) calculating neighboring diffusion-weighted volume correlation and excluding outliers^69^, (2) checking the alignment quality to the connectometry template, and (3) checking for outliers in the connectometry database.

### Group connectometry

Group connectometry was performed both between groups and within groups, to test for categorical disease effects, and to estimate behavioral relevance for connectivity effects seen in CHD, respectively. Connectometry is a statistical approach for investigating local connectivity. First, for each fiber direction within voxels, QA is extracted, and a test statistic is generated for the variable of interest. A t-threshold is then set to perform tractography and generate tracks that are correlated with the variable. Permutation testing is then performed to generate null test statistics then tracks, to which the empirical tracks are compared based on a set length threshold. From this null length distribution, a false discovery rate (FDR) corrected p-value is calculated^57^. Additionally, topology-informed tract pruning (TIP) is performed on the results to control for spurious fibers^70^.

For the between-group analysis, a nonparametric Spearman partial correlation was used to analyze the effect of group on normalized QA at each SDF while controlling for sex. A t-threshold of 3.0 was used to select local connectomes, meaning that only those tracts with a moderate to strong relationship with group were included in hypothesis testing. Two iterations of topology-informed pruning were used to filter tracts^69^. A total of 4000 permutations were applied to group label to generate the null length distribution used to calculate false discovery rate (FDR < 0.05). The length threshold for tracts was set at 40 mm (20 voxels). Within-group connectometry was performed with the same parameters, except for an increase to 3 iterations of tract pruning and an increase of t-threshold to 3.5 to better control false positives, necessary with reduced sample size. Neuropsychological performance included in within-group analyses were the verbal comprehension index (VCI), Visual Spatial Index (VSI), Working Memory Index (WMI), Fluid Reasoning Index (FRI), Processing Speed Index (PSI), and full-scale IQ (FSIQ) from the WISC-V, and a language composite score consisting of the average of PPVT and EVT standard scores.

### Graph theoretical analyses

We extracted global and local graph theoretical properties for each participant to investigate differences in network topology. First, an MNI-space QA template was nonlinearly warped to the study-specific QA template created previously. Then, the warp was applied to the Craddock 200-unit random parcellation scheme^71^ to obtain the parcels in subject space for network analyses. Tracking parameters were: step size of 1 voxel, turning angle of 60°, tracking threshold of 0.6*Ostu’s threshold based on normalized QA values, and 2 iterations of topology-informed pruning. Connectivity matrices were weighted by normalized QA. *t*-tests were performed between groups for network transitivity, global efficiency, and small-worldness.

Hub distributions were also compared between groups. To extract hubs, weighted eigenvector centrality (EC) was calculated for all nodes for each participant then standardized (converted to z-score). Nodes were binarized within each subject based on a z-value > 2 and then summed within each group. Nodes that had a z-value > 2 in 4 or more participants were considered a hub for that group. Since the sample size for our control cohort was small (N = 7), we performed a supplemental analysis on a comparable dataset of controls to assess generalizability of these findings. Details of this analysis can be found in the supplementary material.

## Results

### Neuropsychological testing and demographics

A total of 16 participants (9 CHD, 7 TPC) contributed data to the final analyses (2 CHD and 2 TPC were excluded due to scanner artifacts, and 2 CHD and 2 TPC were excluded for having average QA values more than 2 standard deviations away from the rest of the cohort, 1 TPC was excluded for having difficulty during the neuropsychological testing, and 1 TPC excluded for having incomplete dMRI data). Table 1 shows a comparison of demographic information between the two groups, in addition to specific details of the CHD cohort. There was a significant difference between groups on standardized FSIQ (*t*(15) = − 5.32, *p* < 0.001, M_TPC_ = 134.88, M_CHD_ = 93.33). Fisher’s exact tests revealed no significant differences between groups in sex, handedness, race, ethnicity, parental level of education, or household income (*p* > 0.05). Means, standard deviations, and counts for each group for all neuropsychological and demographic variables are presented in Table 2.

**Table 1:** Demographic and clinical variables of CHD and TPC groups.

	CHD (n = 9)	TPC (n = 7)
**Demographics and cognitive ability**		
Sex		
Male	6	4
Female	3	3
Age (years)		
Range	6.8–11.0	6.4–11.7
Median	8.8	8.5
**Birth characteristics**		
Gestation (weeks)		
Range	36.0–40.0	Unavailable; all > 37 and < 42 weeks
Median	39	
Birth weight (kg)		
Range	2.240–3.640	
Median	2.807	
Mean (SD)	2.973 (0.45)	
Head circumference at birth (cm)		
Range	30.5–35.5	
Median	32.5	
Mean (SD)	32.9 (1.8)	
Length at birth (cm)		
Range	45.0–53.3	
Median	47.5	
Mean (SD)	48.6 (2.6)	
**Cardiac, surgical, and medical characteristics**		
Cardiac diagnoses		
Double outlet right ventricle with left-sided hypoplasia	1	
Double outlet right ventricle with pulmonary outflow obstruction	1	
Double inlet left ventricle	1	
Pulmonary atresia with intact ventricular septum	2	
Single ventricle, otherwise not classified	1	
Tricuspid atresia	2	
Unbalanced AV canal	1	
Stage I palliation		
None	2	
Norwood with BTT shunt	1	
BTT shunt (without CPB)	5	
Central shunt (with CPB)	1	
Hospital LOS after surgery (days)		
Range	6–33	
Median	14	
Mean (SD)	16.9 (8.9)	
Cardiac arrest/CPR	0	
ECMO	0	
Stage 2 palliation surgery		
Age at surgery (days)		
Range	79–263	
Median	152	
Mean (SD)	164 (59)	
Hospital LOS after surgery (days)		
Range	4–26	
Median	6.5	
Mean (SD)	10.3 (7.5)	
Cardiac arrest/CPR	0	
ECMO	0	
Fontan surgery		
Age at surgery (years)		
Range	2.9–5.7	
Median	4.4	
Mean (SD)	4.3 (0.8)	
Hospital LOS after surgery (days)		
Range	5–72	
Median	7	
Mean (SD)	17.2 (22.1)	
Cardiac arrest/CPR	0	
ECMO	0	
Syndromes/associations		
None	5	
Heterotaxy	3	
VATER	1	
Other major organ system anomalies		
None	2	
Musculoskeletal	1	
Renal/genitourinary	2	

*Note: LOS =* *Length of stay.*

**Table 2:** Neuropsychological assessment variables for CHD and TPC groups.

	CHD (N = 9)	TPC (N = 7)	Overall (N = 16)
**Standardized VCI**			
Mean (SD)	101 (11.0)	106 (18.0)	103 (14.2)
Median [Min, Max]	103 [84.0, 118]	106 [86.0, 133]	103 [84.0, 133]
**Standardized VSI**			
Mean (SD)	93.8 (13.9)	98.1 (15.8)	95.7 (14.4)
Median [Min, Max]	92.0 [72.0, 111]	94.0 [81.0, 129]	93.0 [72.0, 129]
**Standardized FRI**			
Mean (SD)	98.9 (11.7)	107 (14.9)	102 (13.3)
Median [Min, Max]	94.0 [88.0, 123]	109 [79.0, 128]	102 [79.0, 128]
**Standardized WMI**			
Mean (SD)	101 (37.4)	97.7 (15.6)	99.4 (29.1)
Median [Min, Max]	85.0 [74.0, 193]	91.0 [85.0, 125]	88.0 [74.0, 193]
**Standardized PSI**			
Mean (SD)	94.0 (14.6)	98.6 (9.47)	96.0 (12.5)
Median [Min, Max]	95.0 [69.0, 111]	95.0 [89.0, 114]	95.0 [69.0, 114]
**Standardized FSIQ**			
Mean (SD)	93.3 (13.1)	131 (17.2)	110 (24.3)
Median [Min, Max]	95.0 [74.0, 118]	130 [106, 160]	105 [74.0, 160]

### Group connectometry: between groups

**Figure 1:**
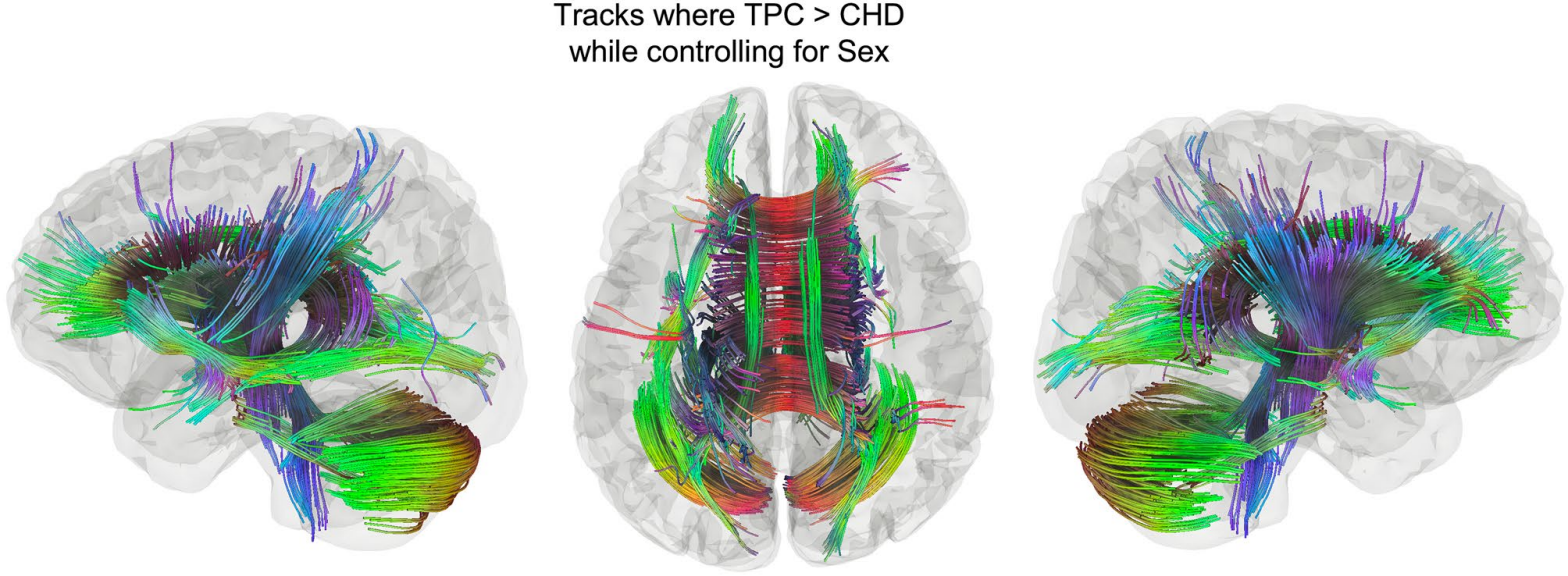
Tracks where TPC > CHD while controlling for sex. This contains much of the white matter, including bilateral inferior fronto-occipital fasciculi (IFOF), anterior and posterior aspects of the body of the corpus callosum (CC), bilateral cinguli, bilateral cerebellar tracts, and portions of bilateral corticospinal tracts (FDR = 0.0056).

Group connectometry revealed multiple tracts where CHD had significantly decreased white matter connectivity compared to TPC while controlling for sex (Fig. 1, *FDR* = 0.0056). Supplementary Fig. 1 shows a box plot of the normalized QA values by group. FSIQ was included in a subsequent model, given that there was a significant difference between groups, to calculate partial correlations. There were no significant effects of group while controlling for FSIQ and sex and no significant effects of FSIQ when controlling for group and sex. This is likely due to the small sample size and by collinearity between FSIQ and group.

### Group connectometry: within groups

**Figure 2:**
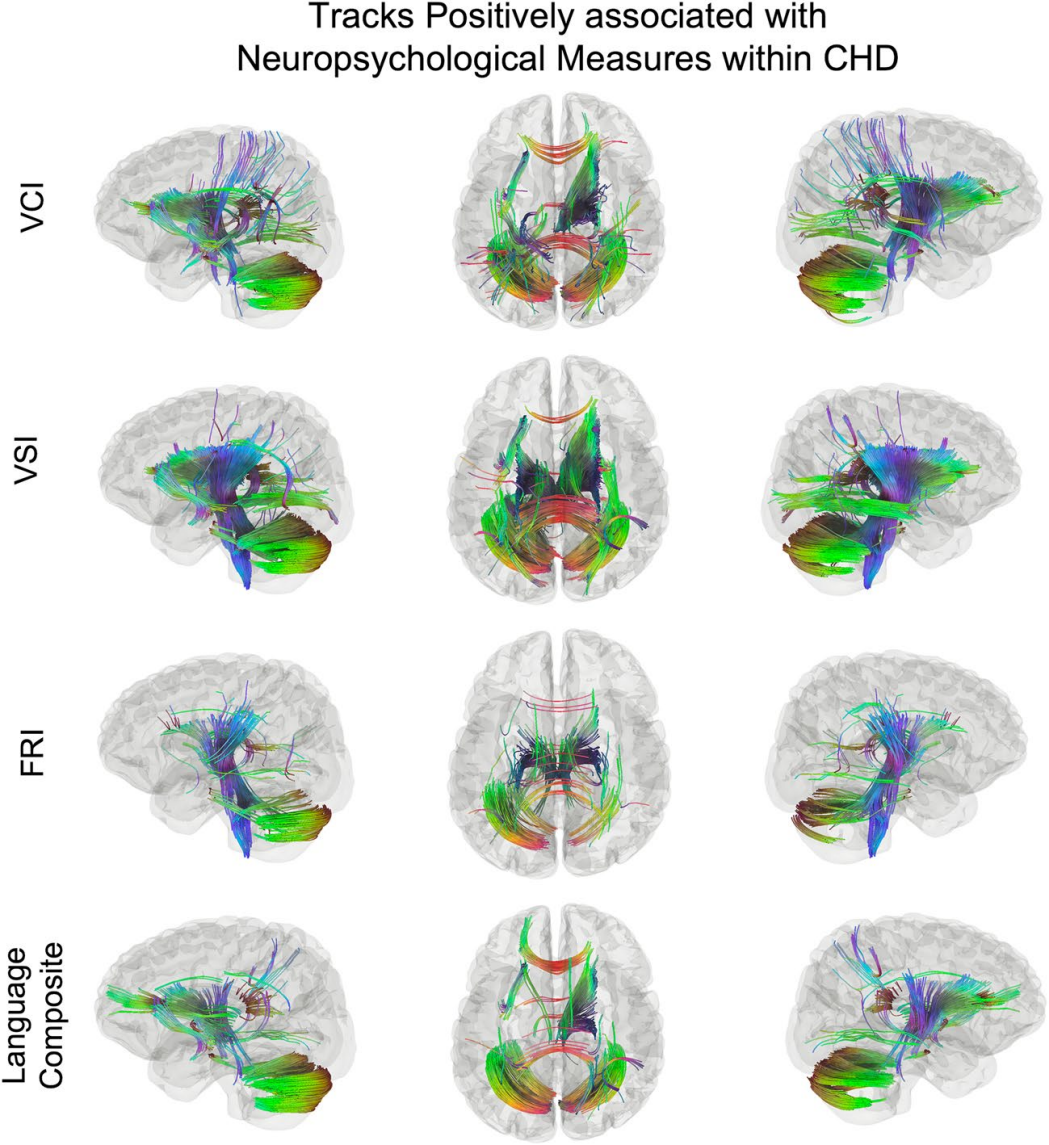
Tracks positively associated with VCI (FDR = 0.029), VSI (FDR = 0.038), FRI (FDR = 0.019), and Language Composite (FDR = 0.033). Tracts associated with VCI include forceps minor and splenium of the CC, bilateral cerebellar tracts, left UF, and bilateral corticothalamic tracts (top row). Tracts correlated with VSI include bilateral CST, left posterior AF, and bilateral posterior portions of the IFOF (second row). Unique tracts related to FRI include bilateral cinguli and tempro-parietal tracts (third row). Tracts associated with Language composite (last row) are nearly identical to those related to VCI (first row).

Within-group analyses for the CHD group showed significant *positive* associations of white matter connectivity with VCI (*FDR* = 0.029), VSI (*FDR* = 0.038), FRI (*FDR* = 0.013), and Language Composite (*FDR* = 0.033; Fig. 2). Within the widespread associations for each of these measures, there are notable findings. First, the pattern of significant tracks is nearly identical between VCI and Language Composite (Fig. 2, first row and last row). Second, there is a clear distinction between VSI and FRI (Fig. 2, second row and third row), in which VSI is associated with motor pathways, cerebellar pathways, and corticothalamic pathways, while FRI is associated with bilateral cingulum and parietal tracts. Third, associations with WMI are the most widespread and involve nearly the entire medullary center (Fig. 2, fourth row). Finally, all results contain bilateral tracts, suggesting the importance of interhemispheric involvement for greater performance. For all within-group results, results have also been presented at more conservative thresholds of *FDR* < 0.025 (Supp. Fig. 3) and *FDR* < 0.01 (Supp. Fig. 4).

Within-group analyses for TPC revealed no significant associations between white matter connectivity and the neuropsychological measures (all *FDR* > 0.05).

### Graph theoretical analyses

**Figure 3:**
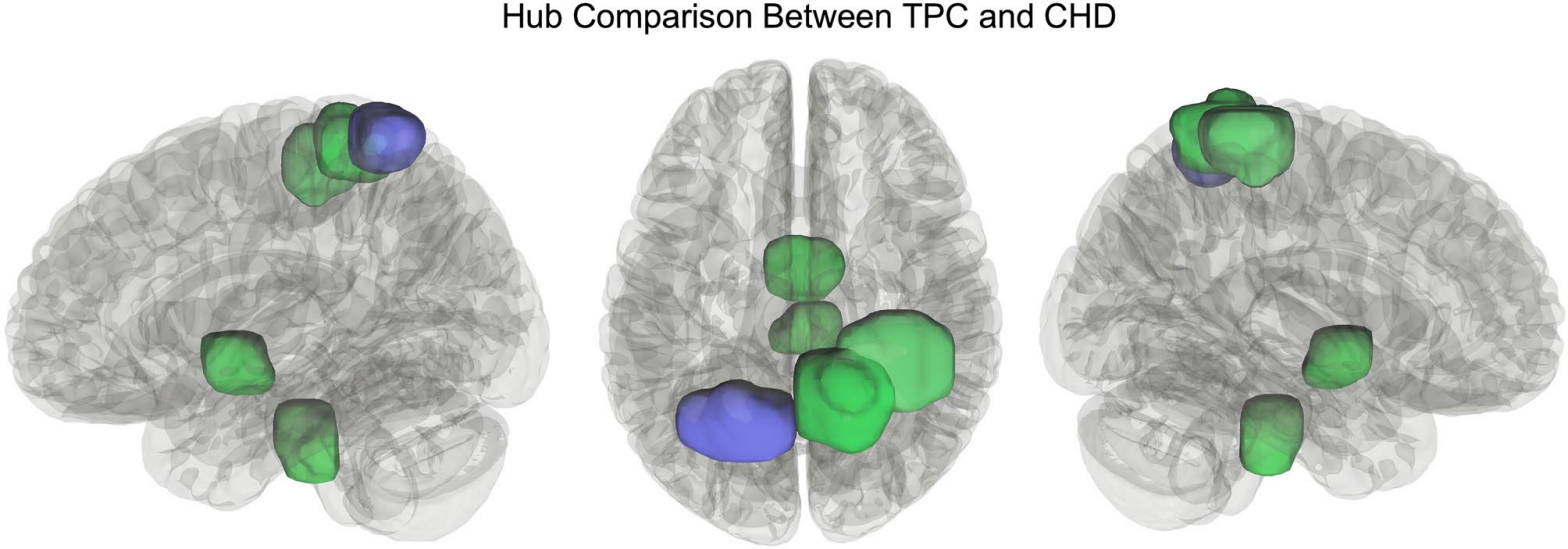
Results of hub analyses between the two groups. Green hubs are those shared by both groups (brain stem, midbrain, and two regions of the right superior parietal lobule). Purple hubs are those only resolved in TPC (1 additional region in the left superior parietal lobule).

There were no significant differences between groups for any of the global network measures. However, hub distribution was qualitatively different between groups. Four hubs were resolved in the CHD group and 7 hubs in the TPC group. Notably, the 4 hubs in the CHD group (brain stem, midbrain, and two regions in the right superior parietal lobule) were also resolved in the TPC group, along with 3 additional hubs (right superior parietal lobule, two regions in left superior parietal lobule; Fig. 3).

## Discussion

In our study of white matter connectivity using recent diffusion imaging analysis methods in early school-age children with CHD, we show: (1) CHD patients had extensive decreases in white matter connectivity compared to TPC, (2) there were several distinct positive associations between QA and neuropsychological measures within CHD patients, and (3) CHD patients had a similar, but less extensive and bilateral hub distribution, than TPC. These results are consistent with previous studies indicating widespread decreased FA in adults and adolescents with CHD and decreases in FA related to worse performance on neuropsychological tests^27^. The current study expands these findings by: (1) investigating a unique, novel cohort of developing children with CHD (survivors of single ventricle palliation) that has not been studied previously using higher-order diffusion analyses; (2) observing white matter connectivity correlations with a comprehensive neuropsychological battery; and (3) performing a hub analysis to reveal differences in network topology in the CHD cohort.

Previous structural brain imaging studies of patients with CHD have largely included children under 2 years in age or adolescent and adult patients. In a study of children at 2 years of age, researchers found significantly decreased intracranial volumes in an array of areas post-Fontan compared to healthy controls, despite the CHD group performing comparably to normative data on the Bayley-III^21^. These findings are very similar to the current work, as the average FSIQ in the CHD was within one standard deviation of the normative average (i.e., 100 ± 15) even though there were widespread decreases in white matter connectivity compared to TPC.

One of the few diffusion imaging studies on middle childhood post-Fontan (10–19 years) used traditional DTI methods to show extensive mean FA decreases and mean MD increases in children with CHD versus healthy controls. Additionally, there were also numerous associations between FSIQ and processing speed and mean FA within several white matter tracts within the CHD group. In the control group, there were no associations between white matter metrics and neuropsychological performance^34^. These results are highly consistent with our findings in a younger cohort (6–11 years) and using more advanced diffusion imaging methods that have distinct advantages (i.e., whole brain analysis, better ability to capture crossing fibers).

In adolescents and adults with a Fontan circulation, DTI studies have shown cognitive impairment and related white matter injury, mostly in deeper white matter tracts^72^. Such adolescent and adult studies of patients with CHD show that, even though CHD patients often perform close to the norm on many cognitive assessments, the results of structural brain injury suffered early in life are chronic, especially myelin changes likely caused by hypoxia-ischemia^73^. Studies highlighting these life-long consequences of CHD following surgical repair could have a significant impact on public health, as many children with CHD are not referred for developmental monitoring or therapies after discharge, despite performing worse than controls and even children born very prematurely (who receive more developmental monitoring and therapies through standardized early intervention programs)^30^.

Finally, no previous studies were found discussing graph theoretical analyses at any stage in development in patients with CHD requiring staged single ventricle palliation. A recent systematic review found 2 papers that used graph theoretical analyses of DTI data in adolescents and young adults with D-transposition of the great arteries who underwent the arterial switch procedure. These studies found topological differences based on global graph theoretical measures (i.e., global efficiency, small-worldness, cost, etc.) between CHD patients and healthy controls that were correlated with worse cognitive performance. However, as the review correctly points out, these global graph measures are highly intercorrelated and these previous studies did not appropriately correct for multiple comparisons^55^. Thus, our results are unique and contribute to the extant body of literature.

The hub analysis presented here did show an interesting pattern. Hub distribution was the same for 4 of the hubs resolved for both groups. In addition to these, TPC had 3 additional hubs, suggesting delayed or arrested network maturation in CHD, in view of our previous work which showed an increase in hubs with age^74,75^. Underdevelopment of the three regions that were defined as hubs in TPC (portions of left and right superior parietal lobules) are consistent with previous studies suggesting impaired attention and executive dysfunction in CHD, as these regions are heavily involved in attention and the fronto-parietal connections that are involved in executive functioning and salience^76^. However, the hub analysis performed here was descriptive in nature, so results should be interpreted carefully until future work can be performed to answer this question quantitatively.

The primary limitation of this study is the small sample size. The inclusion and exclusion criteria for the CHD patients were restrictive to keep the sample as homogenous as possible. However, this choice limited the number of patients that could be recruited in the study timeline. Care was taken with analyses to ensure results are presented responsibly. Future studies will include larger sample sizes with similar imaging protocols so the current results can be validated, and our sample can be expanded.

## Conclusion

Overall, our results suggest abnormal white matter maturation in children with CHD who have completed the Fontan single ventricle palliation pathway. This phenotype is consistent with previous studies suggesting early white matter injury and delayed myelination from damaged pre-oligodendrocytes due to hypoxia-ischemia^19^. While the CHD patients in our study performed close to the normative average, the deficits seen in white matter connectivity may reflect increased vulnerability. Our study contributes to the growing body of evidence characterizing unique and differential neurodevelopmental risk in survivors of complex cyanotic congenital heart disease that was previously considered incompatible with life, suggesting a need for increased neurodevelopmental monitoring in this population.

## Supplementary Information

Supplementary Information.

## Data Availability

The datasets generated during and/or analysed during the current study are not publicly available due to grant restrictions but are available from the corresponding author on reasonable request.
