# Supplementary material for: Altered white matter connectivity in children with congenital heart disease with single ventricle physiology
Source: Sci Rep. 2023 Jan 24;13:1318. doi: 10.1038/s41598-023-28634-9 (PMC9873737; doi:10.1038/s41598-023-28634-9)
Supplement: Supplementary file 1 — Supplementary Information. [file 41598_2023_28634_MOESM1_ESM.pdf]

# Altered White Matter Connectivity in Children with Congenital Heart Disease with Single Ventricle Physiology

Brady J. Williamson, PhD[1], Maria Barnes-Davis, MD, PhD[2,3], Jennifer Vannest, PhD[4], Julia Anixt, MD[3,5], Haleh C. Heydarian, MD[6], Lisa Kuan, MD[5], Cameron S. Laue[7], Jayant "Nick" Pratap, MB BChir, FRCA[8], Mark Schapiro, MD[9], Stephanie Y. Tseng, MD[10], Darren S. Kadis, PhD[11,12]

[1] Department of Radiology, University of Cincinnati, OH
[2] Department of Neonatology, Cincinnati Children's Hospital Medical Center, OH
[3] Department of Pediatrics, University of Cincinnati, OH
[4] Department of Communication Sciences and Disorders, University of Cincinnati, Cincinnati, OH
[5] Division of Developmental and Behavioral Pediatrics, Cincinnati Children's Hospital Medical Center, OH
[6] Cardiology, Cincinnati Children's Hospital Medical Center, OH
[7] Department Psychology, Pacific University, OR
[8] Divisions of Cardiac Anesthesia and Cardiac Critical Care Medicine, Department of Anesthesia and Critical Care Medicine & Cardiac Center, Children's Hospital of Philadelphia, Philadelphia, PA
[9] Neurology, Cincinnati Children's Hospital Medical Center, OH
[10] The Heart Center, Nationwide Children's Hospital, Columbus, OH
[11] Neurosciences and Mental Health, Hospital for Sick Children, Toronto, ON, Canada
[12] Department of Physiology, University of Toronto, ON, Canada

**Corresponding Author:**

**Dr. Darren S. Kadis**
Neurosciences and Mental Health
Research Institute, Hospital for Sick Children

686 Bay Street, Toronto ON  M5G 0A4
+1 (416) 813-7654 ext. 308283
darren.kadis@sickkids.ca

## Supplemental Analysis of CMIND Data

***Methods.*** A total of 37 participants, ages 6-11 years, were taken from the Cincinnati MR Imaging of Neurodevelopment (C-MIND) study, which is available on the National Institute of Mental Health National Data Archive (NDAR; https://nda.nih.gov/edit_collection.html?id=2329). We analyzed the High Angular Resoluation Diffusion imaging (HARDI) acquisiton from this protocol for comparison to the healthy controls in the current study (b=3000, 61 directions, 7 interleaved b0 volumes). While not exactly comparable to the current dataset, this was the closest open-source dataset available with the age range included in this study. Data were subjected to the same processing pipeline (see Methods) with one exception. Only data that had already undergone quality control was avaialbe for this dataset. During the quality control process, gradient directions from each dataset were removed if they did not meet the quality threshold. Only datasets with 80% of the data available within the dataset were kept for the final analysis (number of directions range = 49-61). Eigenvector centrality for each node was extracted and z-transformed within each subject. Nodes with z-score > 2 in 18 or more participants were noted as hubs. 18 was chosen to approximate the same ratio as was used in the control group in the current study (~50%).

***Results.*** While the overall number of hubs (6) and lateral distribution (more left than right) were similar to the current study, the locations of the hubs differed. Two of the 6 nodes resolved overlapped with hubs in our control cohort, but the other 4 were gathered around the brainstem and ventral areas of the brain. This is most likely due to the inability of single shell, relatively low directional acquisitions to adequately resolve crossing fibers architecture within voxels. The hubs that were resolved in the current study are connected by tracts proximal to the corpus callosum, corticospinal tracts, and other regions with known high incidence of incoherent fibers.

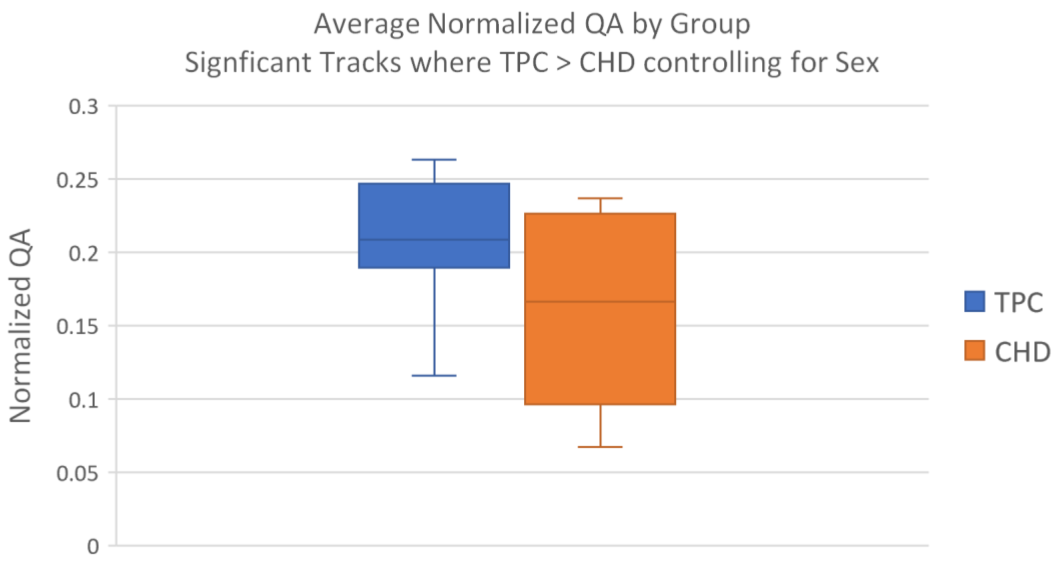


**Supplementary Figure 1.** Normalized quantitative anisotropy (NQA) by group (TPC=blue, CHD=orange) in tracks that had a significant association with group (TPC > CHD) while controlling for sex.

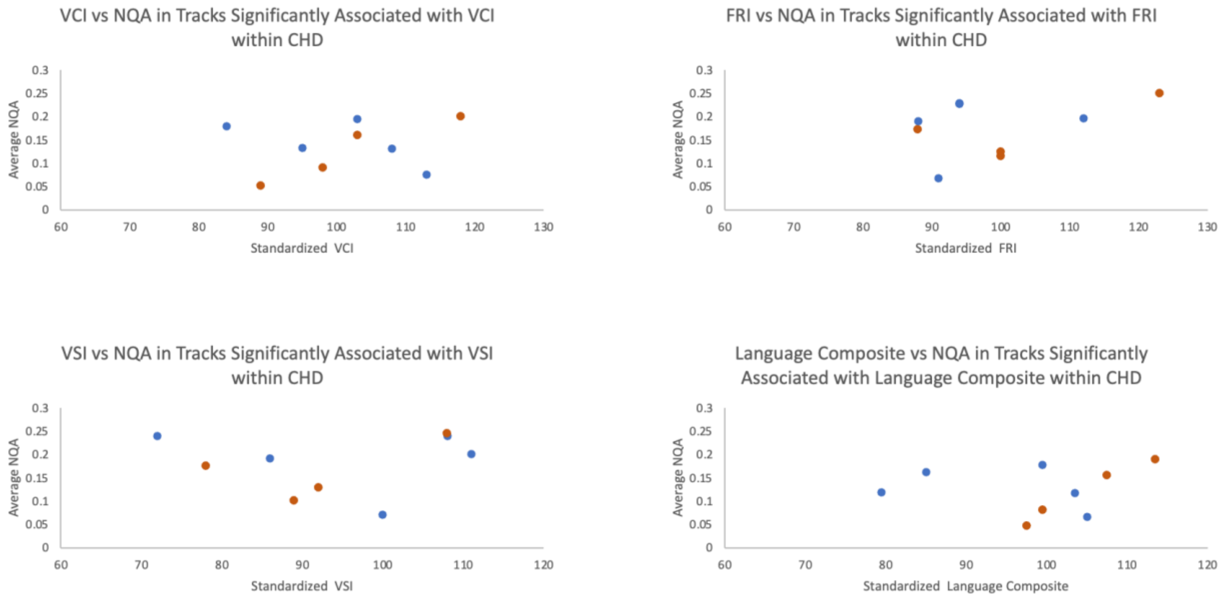


**Supplementary Figure 2.** Plots of neuropsychological variables with average NQA of each participant in the CHD group in tracks that were significant associated with the respective neuropsychological index. Syndromic CHD Patients are highlighted in red.

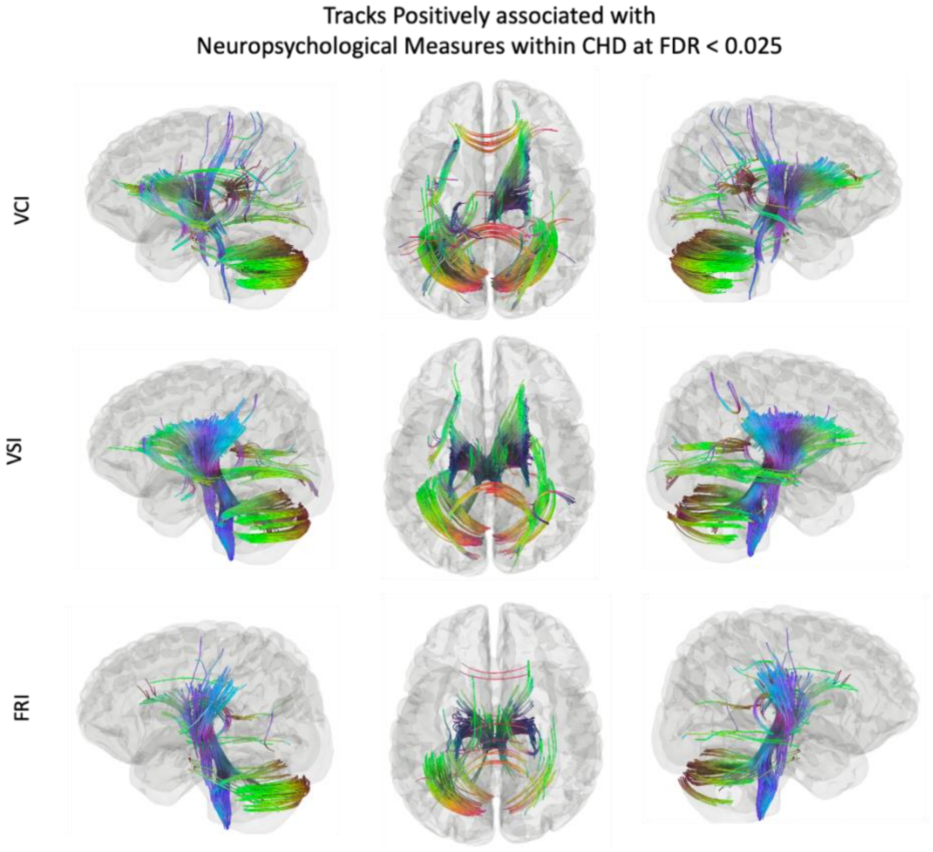


**Supplementary Figure 3.** Tracks associated with neuropsychological variables within the CHD group at *FDR* < 0.025. No tracks were associated with Language Composite at this threshold.

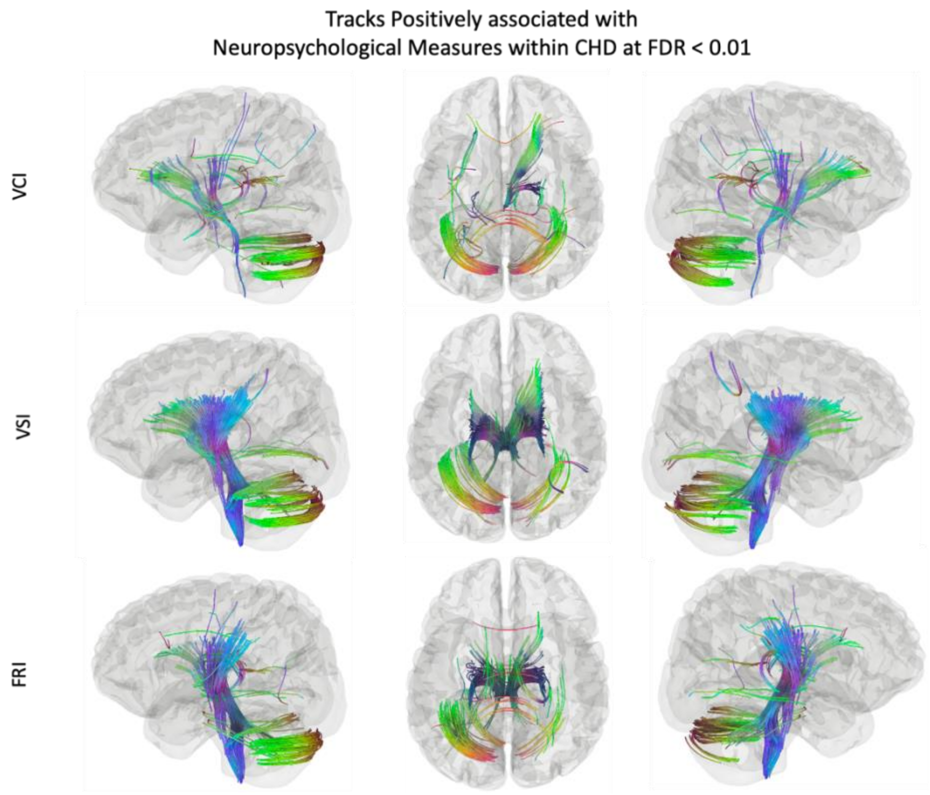


**Supplementary Figure 4.** Tracks associated with neuropsychological variables within the CHD group at *FDR* < 0.01. No tracks were associated with Language Composite at this threshold.

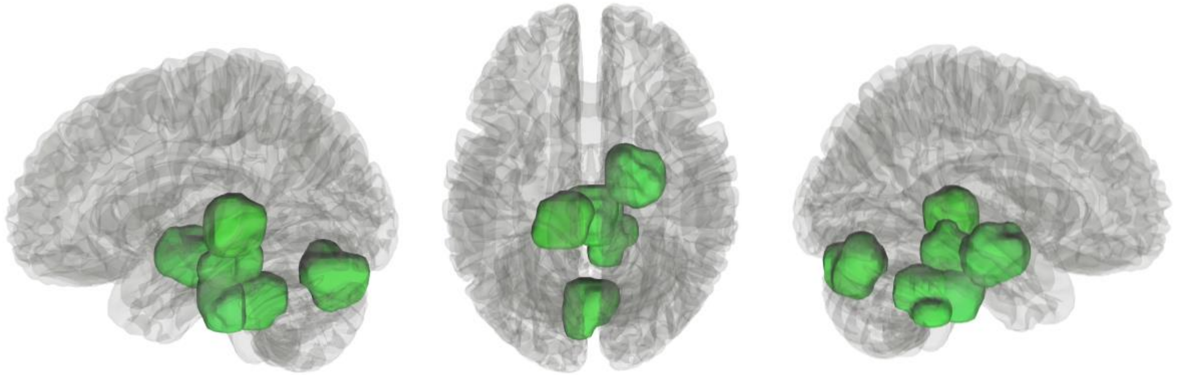

**Supplementary Figure 5.** Hub locations of the CMIND dataset. While the overall number of hubs (6) and lateral distribution (more left than right) is similar to the findings in our current control sample, there were only 2 hubs with direct overlap. This is likely due to the difference in diffusion imaging protocols. The CMIND dataset only acquired a single shell (b=3000) dataset with 61 unique directions, whereas the current study employed a multishell, high directional acquisition better suited to resolve incoherent fibers within voxels.
